# Loneliness in prostate cancer survivors

**DOI:** 10.1007/s00432-026-06539-0

**Published:** 2026-06-14

**Authors:** Lukas Lunger, Maximilian Schwoy, Andreas Dinkel, Valentin H. Meissner, Stefan Schiele, Matthias Jahnen, Jürgen E. Gschwend, Kathleen Herkommer

**Affiliations:** 1 Department of Urology, Klinikum rechts der Isar, School of Medicine and Health, Technical University of Munich, Munich, Germany; 2 Department of Psychosomatic Medicine and Psychotherapy, Klinikum rechts der Isar, School of Medicine and Health, Technical University of Munich, Munich, Germany

**Keywords:** Loneliness, Radical prostatectomy, Prostate cancer, Frailty, Depression, Anxiety

## Abstract

**Background:**

Loneliness in cancer survivors is associated with a variety of negative consequences to physical and mental health. Research regarding the impact of loneliness on health-related quality of life and mental health outcomes in prostate cancer (PC) survivors is scarce. The current study aimed to investigate prevalence and determinants of loneliness in long-term PC survivors following radical prostatectomy (RP).

**Methods:**

A total of 3127 PC survivors (mean age 79.5 years, SD 6.4) from the multicenter German Familial PC Database returned the study questionnaires during the COVID-19 pandemic, after an average of 17.4 years (SD = 3.8) following RP. Loneliness was assessed using a single item measure. Validated self-reporting questionnaires were used to assess anxiety and depressive symptoms, health related quality of life, and frailty. Descriptive statistics, chi-square tests, and logistic regression analysis were conducted to examine the prevalence, correlates, and determinants of loneliness in PC patients.

**Results:**

Overall, 16.6% of patients reported at least some degree of loneliness. Partnership status, depression, and anxiety were significantly associated with loneliness. Individuals living in a partnership experienced lower levels of loneliness (*p* < 0.001), while higher levels of depression and anxiety were associated with increased loneliness (*p* < 0.001). In multivariable logistic regression analysis, having a steady partnership was associated with a significantly lower risk of loneliness (OR 0.18, 95%CI [0.13–0.24]). Symptoms of depression (OR 1.49, 95%CI [1.31–1.71]) or anxiety (OR 1.29, 95%CI [1.13–1.47]) or being frail (OR 3.97, 95%CI [2.99–5.28]) were independently associated with an increased risk of being lonely.

**Conclusions:**

Nearly one in five PC survivors experienced some to severe loneliness in this study, with notable associations with partnership status, symptoms of depression or anxiety and frailty. The findings emphasize the importance of addressing loneliness as part of comprehensive care for PC survivors.

## Introduction

After radical prostatectomy (RP), prostate cancer (PC) survivors may be at risk of significant negative consequences to their physical health (e.g., urinary incontinence, bowel symptoms, erectile dysfunction (Mottet et al. 2022). Additionally, PC survivors may experience emotional and psychological challenges, including treatment decision regret and low quality of life (Hoffman et al. 2017, Baunacke et al. 2020, Meissner et al. 2023), prostate specific antigen (PSA) and PC anxiety (Meissner et al. 2017), depression, general anxiety or fear of cancer recurrence (Meissner et al. 2021). Advances in cancer screening and early detection have led to an increasing number of long-term PC survivors in industrialized countries (Mottet et al. 2022), mandating attention not only regarding survival, but also aspects negatively impacting long-term quality of life. Loneliness as an important determinant of quality of life is still largely unexplored among patients with cancer and PC in particular.

Loneliness is a growing public health concern and is described as a subjective, distressing emotional state that arises when individuals perceive a discrepancy between their desired and actual social connections (Peplau et al. 1982). Figures vary by study populations, age groups included and the measures chosen to assess loneliness (Ong et al. 2016). Previous studies found that being exposed to physical and mental life stressors predicts loneliness (Cacioppo et al. 2010, Hensley et al. 2012). Additionally, social integration among PC survivors was found to be linked to fewer depressive symptoms and improved psychosocial well-being (Chen et al. 2024). Further, loneliness is independently associated with increased morbidity and mortality (Heinrich and Gullone 2006, Friedler et al. 2015, Holt-Lunstad et al. 2015, Wu et al. 2020, Wang et al. 2023, Elovainio et al. 2021), depression (Victor and Yang 2012), suicidality, low quality of life, low resilience (Heinrich and Gullone 2006, Zebhauser et al. 2014), and frailty (Hoogendijk et al. 2016, Herrera-Badilla et al. 2015), both in the general population and patients with cancer (Wells and Kelly 2008, Deckx et al. 2014). Importantly, loneliness may be both a consequence of health-related stressors and a determinant of subsequent adverse health outcomes. However, knowledge on loneliness in cancer patients is limited and generally overlooked, especially in PC survivorship (Wells and Kelly 2008, Rosedale 2009, Brennan et al. 2012, Rokach et al. 2013, Sahin and Tan 2012, Sevil et al. 2006).

PC survivors may be at risk of being lonely due to (1) exposure to significant life stressors associated with PC (e.g. risk of poor functional/oncological outcomes, chronic treatment decision regret, fear of cancer recurrence, PSA/PC-anxiety, depression or general anxiety), (2) frailty and functional impairment following treatment and ageing, and (3) older age and related life circumstances (e.g., widowhood and shrinking social networks).

Thus, the aim of this study was to examine the prevalence and determinants of loneliness in PC survivors to gain a comprehensive understanding of the unique challenges faced by these individuals. To our knowledge, this is the first analysis of loneliness among long-term PC survivors and comprises the largest number of cases with an average follow-up of 17 years.

## Methods

### Study population and procedures

For this study, PC survivors registered in the prospective German Familial Prostate Cancer database were contacted. The database is updated annually via questionnaires including sociodemographic, clinicopathological, and psychological characteristics. Individual consent was obtained from all participants; the study was performed in line with the principles of the Declaration of Helsinki and was approved by the ethical review committee of the Technical University of Munich. Detailed database descriptions were previously provided (Meissner et al. 2021, Meissner et al. 2020).

PC survivors were eligible for this study if they (1) had RP as first-line treatment and (2) returned the questionnaires including the item on loneliness during the COVID-19 pandemic, more specifically between November 2021 and August 2022. A total of 5526 PC survivors participated in the study.

Non-responders were defined as patients who did not return the questionnaires including the item “loneliness” at the designated follow-up time point. To assess potential biases or differences between responders and non-responders, sociodemographic and clinical characteristics (age at surgery, organ confined disease at RP, level of education, children) were compared.

### Measures

For this study, the following sociodemographic features were included: age at survey (years), partnership status (steady partnership yes versus no), level of education (low, intermediate, high) and having children (yes versus no). The subjective economic situation was assessed through a five-point Likert scale response, ranging from poor to very good, and subsequently recoded into two categories: good and unsatisfactory.

Clinicopathological characteristics included years since RP, positive PC family history (defined as at least one first-degree relative with PC), prostate-specific antigen (PSA) level at diagnosis (ng/ml), organ-confined disease at RP (≤ pT2c and pN0), presence of biochemical recurrence (defined as rising PSA value ≥ 0.2 ng/ml since RP), current therapy (none versus androgen deprivation therapy versus other), secondary cancers in life, covid-19 history (no versus yes (without hospitalization) versus yes (with hospitalization)).

Loneliness was assessed using a validated single item (“I am frequently alone /have few contacts”) as previously described (Beutel et al. 2017, Aßmann et al. 2024). The degree of loneliness was estimated as follows: PC survivors were asked to choose between 0 = no, does not apply, 1 = yes, but I do not suffer from it, 2 = yes, and I suffer slightly, 3 = yes, and I suffer moderately, and 4 = yes, I suffer severely from loneliness. Thus, the item captures aspects of social contact and the perceived burden associated with it. The response categories 0 and 1 were recoded as “no loneliness”.

Depressive and anxiety symptoms were assessed using the validated Patient Health Questionnaire-4 (PHQ-4), an ultra-brief screening tool consisting of a two-item depression scale (PHQ-2) and a two-item anxiety scale (Generalized Anxiety Disorder 2 (GAD-2). A score ≥ 3 indicates clinical levels of depression and anxiety, respectively (Meissner et al. 2021, Löwe et al. 2010).

Global quality of life was assessed using items 29 and 30 of the EORTC QLQ-C30 1993. Frailty was assessed using the validated Groningen Frailty Index (GFI) and defined as a score ≥ 4  (Steverink et al. 2001). The GFI is a 15-item questionnaire (score range 0–15) and evaluates four domains: physical, cognitive, social, and psychological.

### Statistical analyses

All data analyses were conducted using SAS 9.4 ((SAS Institute Inc.) Cary, NC, USA). To assess potential biases or differences between responders and non-responders, sociodemographic and clinical characteristics (age at surgery, organ confined disease at RP, level of education, children) were compared between the two groups with chi-square tests for categorical variables, and Wilcoxon–Mann–Whitney tests for continuous variables. For comparisons between groups (no versus slight versus moderate/severe loneliness) by sociodemographic, clinicopathological, and psychological characteristics chi-square tests were computed.

Multivariable logistic regression analyses using the backward elimination method were performed to determine the association of selected sociodemographic, clinicopathological, and psychological parameters with loneliness (recoded as yes [scores ≥ 2] versus no [scores 0 and 1]). Backward elimination was applied to identify independent predictors of loneliness while avoiding overfitting. All variables entered into the initial model were selected a priori based on clinical relevance and literature. Absolute numbers, percentages, means with standard deviations (SD), odds ratios (OR) including 95% confidence intervals (CI) and p-values were reported, with the 0.05 level considered statistically significant.

## Results

Of the 5526 PC survivors enrolled in the trial, 3210 (58.1%) returned the questionnaires, 2117 did not answer within 9 months and 199 had died. A total of 83 patients did not reply to the item on “loneliness” and were therefore not included in this analysis. The comparison of demographic and clinical characteristics between responders and non-responders revealed that non-responders were older at surgery (*p* < 0.001), had more often non-organ confined disease at RP (*p* < 0.001), and a greater proportion of non-responders had no children (*p* < 0.001).

Table 1 illustrates the characteristics of the study population. A total of 3127 study participants (mean age of M = 79.5 years (SD = 6.4) completed the survey after a mean time of M = 17.4 years (SD = 3.8) following RP.

**Table 1:** Patient characteristics (*n* = 3127)

Parameters	Mean (SD)	n	%
*Sociodemographic parameters*			
Age at survey (years)	79.5 (6.4)	3127	
Partnership			
Yes		2654	86.5
No		413	13.5
Level of education			
Low		1168	39.0
Intermediate		512	17.1
High		1312	43.9
Children			
Yes	1.8 (1.0)	2727	88.5
No		353	11.5
Subjective economic situation			
Good		3028	98.2
Unsatisfactory		57	1.8
*Clinicopathological parameters*			
Time since radical prostatectomy (years)	17.4 (3.8)	3127	
Positive prostate cancer family history			
Yes		1242	39.7
No		1885	60.3
Organ confined disease at RP			
Yes		2235	72.0
No		869	28.0
Biochemical recurrence during follow-up			
Yes		1142	36.7
No		1967	63.3
Current therapy			
Androgen deprivation therapy		321	10.2
Other		2	0.1
None		2804	89.7
Second primary cancer			
Yes		422	13.5
No		2705	86.5
Covid-19			
Yes, hospitalization		17	0.6
Yes, no hospitalization		121	3.9
No		2954	95.5
Frailty (GFI)	2.9 (2.4)		
No (<4)		1968	67.2
Yes (≥4)		959	32.8
*Psychological parameters*			
Loneliness			
No		2605	83.4
Yes, I suffer slightly		333	10.6
Yes, I suffer moderately		150	4.8
Yes, I suffer severely		39	1.2
Depression (PHQ-2)	1.0 (1.2)		
No (<3)		2702	89.2
Yes (≥3)		326	10.8
Anxiety (GAD-2)	0.9 (1.2)		
No (<3)		2765	91.7
Yes (≥3)		250	8.3
Global quality of life (percent)	66.2 (19.3)		

*GAD-2 * General anxiety disorder scale-2,* FI* Groningen frailty index,* PHQ-2 * Patient health questionnaire-2,* RP* Radical prostatectomy,* SD* Standard deviation

Overall, 16.6% of PC survivors reported to suffer from loneliness: 10.6% felt slightly, 4.8% moderately, and 1.2% severely burdened by loneliness.

**Fig. 1:**
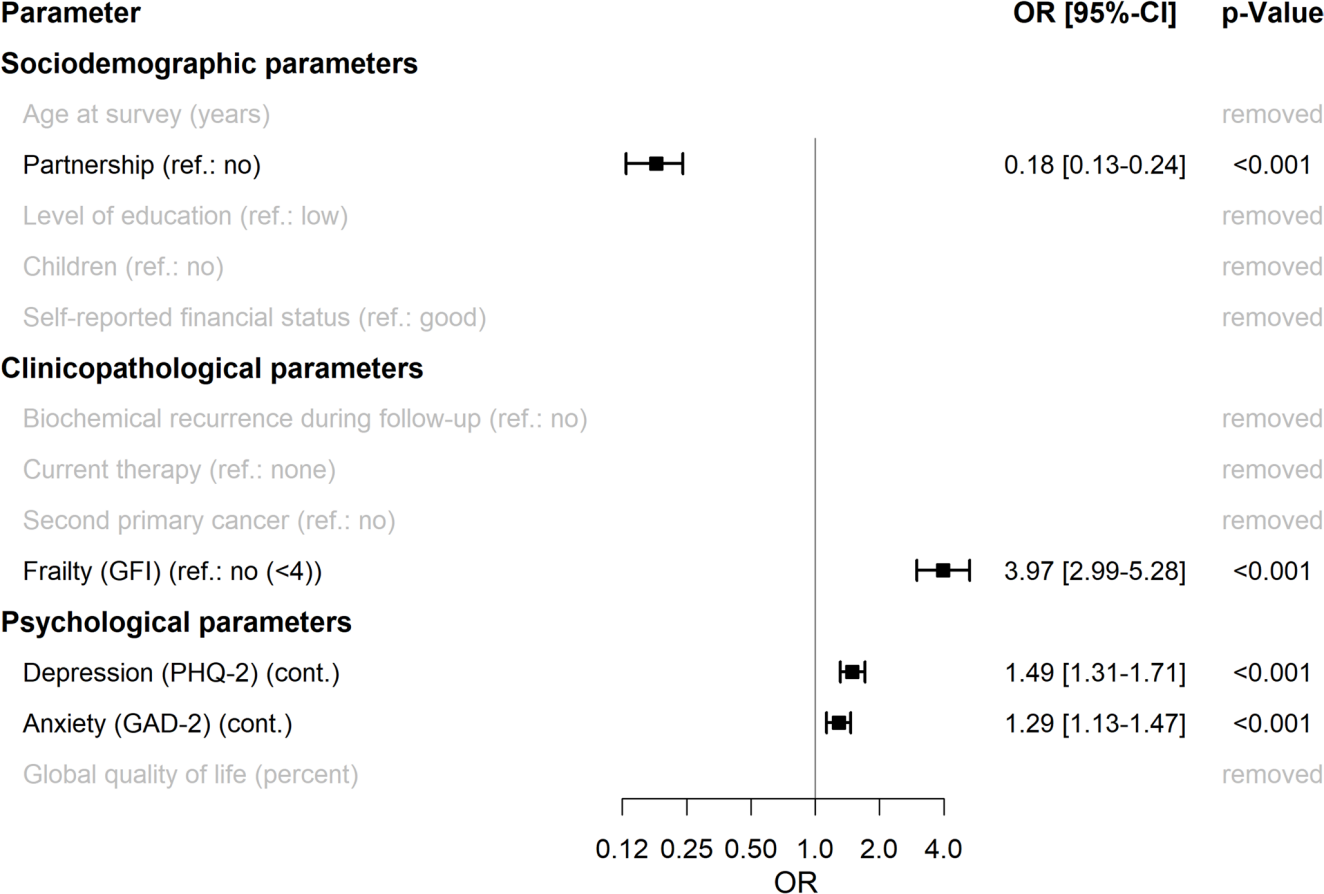
Multivariable logistic regression analysis (backward elimination) to assess the association of sociodemographic, clinicopathological, and psychological parameters with loneliness (recoded as yes [≥ 2] versus no [0 and 1]). Variables excluded during the backward elimination process are shown in grey. Abbreviations: CI = confidence interval; GAD-2 = Generalized Anxiety Disorder 2; OR = odds ratio; PHQ-2 = Patient Health Questionnaire 2; Ref. = reference

Table 2 illustrates the comparison of PC survivors with different degrees of loneliness (none versus slight versus moderate/severe loneliness) based on sociodemographic, clinicopathological, and psychological characteristics. Significant associations with loneliness were observed across several sociodemographic, clinicopathological, and psychological parameters. Age was associated with higher levels of loneliness (*p* < 0.001), and individuals living in a partnership experienced lower levels of loneliness compared to those without a partner (*p* < 0.001). Higher education was linked to lower loneliness (*p* = 0.033). Those reporting a good economic situation had less loneliness compared to those with an unsatisfactory economic situation (*p* < 0.001). Regarding clinicopathological factors, no significant associations were found for time since radical prostatectomy, family history of prostate cancer, disease stage, biochemical recurrence, or current therapy. However, a significant association emerged for second primary cancer (*p* = 0.033). Frailty was significantly associated with loneliness, with higher frailty correlating with increased loneliness (*p* < 0.001). Psychological factors also played a relevant role, with depression and anxiety both linked to higher levels of loneliness (*p* < 0.001). Finally, quality of life was inversely associated with loneliness, with individuals reporting poorer quality of life experiencing higher levels of loneliness (*p* < 0.001). Multivariable analysis (Fig. 1) revealed that after backward elimination four variables were independently associated with loneliness: Being in a steady partnership significantly reduced the risk of experiencing any degree of loneliness among long-term PC survivors by 82% (OR 0.18, 95%CI [0.13–0.24]). Symptoms of depression (OR 1.49, 95%CI [1.31–1.71]) and anxiety (OR 1.29, 95%CI [1.13–1.47]) were both significantly associated with higher risk of loneliness after RP. Being frail (OR 3.97, 95%CI [2.99–5.28]) was associated with a nearly 4-fold higher risk of being lonely.

## Discussion

This study explores the prevalence and determinants of loneliness in long-term PC survivors, providing valuable insights into a largely under-researched area. Our results indicate that nearly one in five PC survivors experience some degree of loneliness, with significant associations found between loneliness and factors such as relationship status, symptoms of depression and anxiety, and frailty. Loneliness is a well-established concern in cancer survivorship, but research specifically focusing on PC survivors is sparse. Our findings are consistent with previous studies reporting high rates of loneliness in cancer survivors, particularly those with ongoing mental health challenges, frailty, and limited social support.

We found that nearly one in five (16.6%) PC survivors reported experiencing some degree of loneliness. The prevalence of loneliness (any degree) among PC survivors observed in this study was either lower (16.6% versus 53% (Miaskowski et al. 2021), 52.2% (Howden et al. 2022)) or nearly comparable (16.6% versus 25% (Dahill et al. 2020)) to other studies investigating loneliness and isolation in oncology patients. Among non-oncology adults, loneliness estimates range from 25 to 29% (Ong et al. 2016). However, a comparison of previous studies to the current results is difficult, given that the prevalence of loneliness may vary as a function of (a) the measures used to assess loneliness, (b) the population studied, (c) the age group investigated and (d) the sample sizes considered (Ong et al. 2016). Also, especially the investigated time-period of the study might be of relevance: both studies estimating loneliness at ~ 50% assessed loneliness among oncology patients throughout the beginning of the COVID-19 pandemic. The COVID-19 pandemic certainly could have boosted the rates of patients expressing loneliness, given that the pandemic required social distancing strategies crucial to limiting the spread of the virus (Aßmann et al. 2024). Although loneliness and social isolation are not equal, both frequently co-occur and have a detrimental effect on physical and emotional well-being (Hwang et al. 2020). Of note, a general population-based analysis based on approximately 5000 German individuals reported similar rates of loneliness (~ 23%) based on the same single-item measure to assess loneliness as was used for this current analysis (Reinwarth et al. 2023). These findings suggest that the COVID-19 pandemic at least did not significantly worsen loneliness among PC survivors in this study compared to the general population during non-pandemic periods. However, it is important to highlight that 89% of prostate cancer survivors in our study were in a relationship, compared to just 60% in the general population. This higher rate of individuals living in a partnership possibly played a key role in helping survivors cope more effectively during the pandemic. A comparison of our results to available data is therefore difficult. Nonetheless, it is evident that loneliness is a prevalent issue in PC survivors that warrants attention. Our study reflects a large cancer survivor population and adds valuable knowledge to the field, given that most available data on loneliness are either based on the general population, geriatric patients or patients actively suffering from cancer in a metastatic setting.

Direct comparison of loneliness prevalence across oncology studies remains difficult because the literature uses a wide range of non-uniform assessment approaches. A recent systematic review of pre-COVID-19 pandemic oncology studies showed that most instruments were used only once, that psychometric information such as test–retest reliability and validity was often lacking, and that the field frequently blurs the distinction between social isolation as an objective lack of contact and loneliness as a subjective perception of insufficient social connection. This is particularly relevant for the present study, because our single-item measure captures aspects of both limited social contact and the perceived burden associated with that state. Accordingly, our prevalence estimates should be interpreted in the context of this broader measurement heterogeneity (Marziliano et al. 2024). 

In this study, key risk factors for loneliness were identified. Notably, survivors living in a steady partnership were significantly less likely to report loneliness, echoing findings from other studies in aging populations. In contrast, those with higher levels of depression, anxiety and frailty had a greater risk of loneliness. Importantly, the average age of the study population was nearly 80 years. Research on age differences in social processes found that older adults may rather report smaller social networks (Cumming and Henry 1961), given that the elderly population may be more likely to be widowed, have friends who had died or live alone (Hobbs 2002, Macunovich et al. 1995). In this context, it is noteworthy that women, on average, tend to maintain broader and more diverse social networks across the lifespan, whereas men’s social connections are more often centered around their partner. This difference may partly explain why men can be particularly vulnerable to loneliness following widowhood or loss of a partner. Thus, one might assume that elderly people and especially elderly PC survivors would be at higher risk of feeling lonely. Of interest, although age was statistically associated with loneliness in unadjusted analyses, the absolute differences between groups were small, and age was not retained as an independent correlate in the multivariable model. This likely reflects overlap with closely related age-associated factors captured in our dataset, particularly frailty and partnership status. Accordingly, this finding should not be interpreted as evidence that age is unrelated to loneliness, but rather that its effect may be reflected by these variables. Also, in this study and in line with previous findings on loneliness in the general population and patients with cancer (Miaskowski et al. 2021, Howden et al. 2022, Hawkley and Capitanio 2015), living in a relationship was associated with lower risk of being lonely, while having children was not. First, it is plausible to speculate that while older PC survivors may have objectively smaller and fewer social networks compared to younger individuals, these limited social networks may hold more significance and relevance in their lives and contribute to greater resilience when facing feelings of isolation and loneliness. Thus, the quality and depth of social connections may outweigh the sheer quantity of connections, highlighting the importance of meaningful relationships in mitigating the negative effects of social isolation and loneliness among older PC survivors. Surprisingly, having children may not necessarily serve as a safeguard against loneliness, as the mere presence of children does not automatically guarantee fulfilling and meaningful relationships. Taken together, these results highlight the importance for healthcare professionals to encourage PC survivors, irrespective of their age, to keep strong bonds given the favorable effect on their psychosocial quality of life (Chen et al. 2024). Where available, structured referral to peer/support groups and community-based activities may help translate this into practice. Engaging in support groups specifically designed for cancer survivors could be valuable to PC survivors at an increased risk of experiencing loneliness.

Second, the results of this study indicate a significant association between elevated symptoms of depression or anxiety and a higher risk of loneliness among PC survivors. These findings reaffirm the well-established association between anxiety, depression, and loneliness (Ong et al. 2016, Cacioppo et al. 2006, Mushtaq et al. 2014, Gallagher et al. 2021) and suggest that long-term PC survivors who are dealing with depression or anxiety are also more likely to be experiencing heightened feelings of loneliness. This could be attributed to the emotional and psychological hurdles they experience throughout their survivorship journey. Recognizing this link is crucial for healthcare professionals in providing comprehensive care to PC survivors. Interventions aimed at addressing mental health concerns, such as referrals for counseling, psychotherapy, or pharmacological treatments, should be integrated into survivorship care plans. Moreover, fostering a supportive environment during follow-up visits where survivors feel comfortable discussing their emotional well-being and providing appropriate resources for mental health support can contribute to mitigate loneliness and improving the overall quality of life for these individuals.

Third, the results of this study show that long-term prostate cancer survivors experiencing frailty are at nearly four times higher risk to feel lonely, which underscores previous findings on the association between frailty and loneliness (Gale et al. 2018, Sha et al. 2022). These observations highlight the importance of healthcare professionals taking a proactive approach to address frailty in PC survivors. By providing tailored medical care, implementing comprehensive support services, and employing multidisciplinary interventions, healthcare providers can effectively address the physical and functional limitations associated with frailty (Dent et al. 2019). Thus, by enhancing the overall well-being and functional capacity of these individuals, interventions focused on reducing frailty can foster social engagement, enhance social support networks, and ultimately contribute to a reduced sense of loneliness. Additionally, interventions targeting frailty could improve not only physical health, but also social connectedness, thus mitigating loneliness. It is important to note that while our study found a nearly four-fold increased likelihood of loneliness among frail patients, the strength of the association may differ in other populations due to differences in study design, sample characteristics, and the measures used to assess frailty and loneliness. Previous studies, such as those referenced by Gale et al. and Sha et al. (2018, 2022), also found associations between frailty and loneliness, but the reported strengths of these associations varied, and the methodologies used for assessing frailty and loneliness differed. These differences in measurement and population make direct comparisons challenging, but the consistency of the association across studies suggests that frailty remains an important factor in the experience of loneliness.

Recently, Lim et al. presented a biopsychosocial model of loneliness, which conceptualizes loneliness as arising from an interplay of situational triggers (e.g., life events), predisposing risk factors, and modifiable targets for intervention, differentiating triggers, risk factors, and potential areas for mitigating loneliness. In our study, by chance, the COVID-19 pandemic acted as a trigger (Lim et al. 2020). However, our results did not show an elevated frequency of loneliness compared to available evidence from before the pandemic. Regarding the postulated risk factors, we were available to include some of the many potential fixed (e.g., age, illness characteristics), and modifiable (e.g. mental health) factors. Interestingly, in our study, mainly modifiable factors showed a unique contribution to the experience of burdensome loneliness. This could encourage efforts to develop interventions tailored to the needs of older long-term PC survivors who present with a set of psychosocial risk factors, as evident in our study. In line with recent research, the importance of modifiable factors in addressing loneliness becomes even more apparent. For instance, a higher sense of purpose has been consistently associated with lower levels of concurrent loneliness and protection against the development of loneliness over time (Sutin et al. 2022). This finding, replicated across diverse cohorts from North America, South America, Europe, and the Middle East, suggests that cultivating a sense of purpose may be a valuable target for interventions, particularly among those experiencing psychological distress (Sutin et al. 2022). Additionally, a systematic review of loneliness interventions in cancer survivors highlights the scarcity of tailored interventions but points to the potential for culturally relevant programs to reduce loneliness. These insights emphasize the need for interventions that not only address social isolation but also foster psychological resilience, particularly in older cancer survivors (McElfresh et al. 2021). Beyond the variables available in our dataset, work in chronic illness cohorts suggests that perceived stigma, greater illness intrusiveness, and lower perceived social support are independent predictors of loneliness; future prostate cancer survivorship research should test these domains (e.g., perceived support and stigma related to functional impairments) to better inform targeted interventions (Nicoloro-SantaBarbara et al. 2024). 

Several limitations must be acknowledged. First, the use of a single-item measure for loneliness may not fully capture the complexity of this emotional experience. The item used in this study combines aspects of social isolation (being alone or having few contacts) with the perceived burden of this state and therefore does not clearly distinguish between these related but conceptually distinct constructs. In addition, individuals who are socially connected but still experience subjective loneliness may not be fully captured by this measure, potentially leading to an underestimation of loneliness prevalence. Nonetheless, it is important to note that this measure has been validated and demonstrated to be reliable in previous large-scale research (Beutel et al. 2017, Aßmann et al. 2024). Additionally, our study focused exclusively on PC survivors who underwent RP. While this means our findings may not be directly generalizable to other treatment groups (e.g. definitive radiation) or patients with metastatic disease, it also offers the advantage of providing a large, and homogenous sample. The homogeneity of our sample allows a more precise understanding of loneliness in this specific group of survivors. Another limitation is the non-response rate, with almost half of the contacted survivors not returning the questionnaire. Indeed, non-responders were older at baseline and had a lower education than responders, which might have introduced some bias. However, non-response rates of this magnitude are common in surveys involving older populations and the non-response rate observed in this study is consistent with other works in the field (Evans et al. 2004). Our cohort consisted of prostate cancer survivors who were, on average, around 80 years old at the time of the survey, and older adults often have lower response rates due to factors such as cognitive decline, physical limitations, or lack of interest in participating. Non-participation may also be systematic, with more vulnerable survivors (e.g., poorer health, higher frailty, or greater psychological distress) potentially underrepresented, which could have led to an underestimation of loneliness and its associations. Furthermore, we acknowledge that patients with more advanced disease at RP (e.g., higher Gleason scores or more aggressive disease) may not have been part of the analysis due to progression or mortality, which could have impacted the study’s findings. Nonetheless, we accounted for factors such as current treatment in the logistic regression analysis, and these variables were excluded during backward elimination as they were not independently associated with loneliness. Furthermore, the multivariable model was derived using backward elimination. While all candidate variables were pre-specified, correlations between closely related constructs (e.g., age, frailty, partnership status) may influence which variables are retained. As such, non-retention should not be interpreted as proven lack of relevance. Next, it should be noted that our study was conducted in an industrialized Western country, including only men who were fluent in German. In addition, findings may not fully generalize to female cancer survivors, given known differences in social networks, partnership patterns, and help-seeking behavior. Thus, the role of cultural factors in social connectedness and the relevance of social bonds and gender for adaptational outcomes were not addressed inthis study. Finally, the cross-sectional nature of the study does not allow to draw causal conclusions. Longitudinal studies would be valuable to examine how loneliness evolves over time and how it correlates with other aspects of survivorship, such as quality of life and functional outcomes.

In conclusion, this study provides valuable insights into the prevalence and risk factors associated with loneliness in long-term PC survivors. The findings highlight the importance of addressing psychological and social needs in PC cancer survivors, particularly those at higher risk of loneliness. Healthcare professionals should encourage social support and address frailty as a means of reducing feelings of loneliness in PC survivors. By addressing these needs, healthcare professionals can improve the overall well-being and quality of life of PC survivors.

**Table 2:** Presence and distribution of loneliness (none, slight, severe) acrosssociodemographic, clinicopathological, and psychological parameters

	*Loneliness*						
	None (n=2605)		Slight (n=333)		Moderate/severe (n=189)		*p*-value
	n	%	n	%	n	%	
*Sociodemographic parameters*							
Age at survey (years)							
Mean (SD)	79.4	(6.3)	79.8	(7.1)	80.7	(6.2)	<0.001
Partnership							
Yes	2135	87.2	222	8.4	117	4.4	<0.001
No	236	57.1	109	26.4	68	16.5	
Level of education							
Low	949	81.3	136	11.6	83	7.1	0.033
Intermediate	434	84.8	58	11.3	20	3.9	
High	1114	84.9	128	9.8	70	5.3	
Children							
Yes	2281	83.6	289	10.6	157	5.8	0.344
No	287	81.3	39	11.1	27	7.6	
Subjective economic situation							
Good	2548	84.2	316	10.4	164	5.4	<0.001
Unsatisfactory	22	38.6	12	21.1	23	40.3	
*Clinicopathological parameters*							
Time since RP (years)							
Mean (SD)	17.4	(3.7)	17.6	(3.9)	17.8	(3.8)	0.22
Positive PC family history							
Yes	1014	81.6	149	12.0	79	6.4	0.103
No	1591	84.4	184	9.8	110	5.8	
Organ confined disease at RP							
Yes	1876	83.9	230	10.3	129	5.8	0.403
No	712	81.9	100	11.5	57	6.6	
BR during follow-up							
Yes	952	83.4	114	10.0	76	6.6	0.464
No	1640	83.4	214	10.9	113	5.7	
Current therapy							
Any	257	79.6	40	12.4	26	8.0	0.134
None	2348	83.7	293	10.5	163	5.8	
Second primary cancer							
Yes	335	79.4	51	12.1	36	8.5	0.033
No	2270	83.9	282	10.4	153	5.7	
Frailty (GFI)							
Mean (SD)	2.4	(2.1)	4.9	(2.5)	6.2	(2.6)	<0.001
No (< 4)	1852	94.1	91	4.6	25	1.3	<0.001
Yes (≥ 4)	607	63.3	211	22.0	141	14.7	
*Psychological parameters*							
Depression (PHQ-2)							
Mean (SD)	0.8	(1.1)	0.7	(1.1)	2.8	(1.5)	<0.001
No (< 3)	2373	87.8	245	9.1	84	3.1	<0.001
Yes (≥ 3)	166	50.9	71	21.8	89	27.3	
Anxiety (GAD-2)							
Mean (SD)	0.7	1.07	1.6	1.3	2.5	1.6	<0.001
No (< 3)	2411	87.2	253	9.1	101	3.7	<0.001
Yes (≥ 3)	121	48.4	59	23.6	70	28.0	
Quality of life (%)							
Mean (SD)	68	(18)	57	(18)	46	(21)	<0.001

*BR* Biochemical recurrence,* GAD-2*  General anxiety disorder scale-2,* GFI* Groningen frailty index,* PC* Prostate cancer,* PHQ-2* Patient health questionnaire-2,* RP* Radical prostatectomy,* SD* Standard deviation

## Data Availability

No datasets were generated or analysed during the current study.
